# Supervised clustering for TSPO PET imaging

**DOI:** 10.1007/s00259-021-05309-z

**Published:** 2021-03-29

**Authors:** Julia Schubert, Matteo Tonietto, Federico Turkheimer, Paolo Zanotti-Fregonara, Mattia Veronese

**Affiliations:** 1 Centre for Neuroimaging Sciences, Institute of Psychiatry, Psychology and Neuroscience, King’s College London, London, UK; 2 Université Paris-Saclay, CEA, CNRS, Inserm, BioMaps, Service Hospitalier Frédéric Joliot, Orsay, France; 3 Molecular Imaging Branch, National Institute of Mental Health, National Institutes of Health, Bethesda, MD USA

**Keywords:** TSPO, PET, Supervised clustering, Pseudo-reference region

## Abstract

**Purpose:**

This technical note seeks to act as a practical guide for implementing a supervised clustering algorithm (SVCA) reference region approach and to explain the main strengths and limitations of the technique in the context of 18-kilodalton translocator protein (TSPO) positron emission tomography (PET) studies in experimental medicine.

**Background:**

TSPO PET is the most widely used imaging technique for studying neuroinflammation in vivo in humans. Quantifying neuroinflammation with PET can be a challenging and invasive procedure, especially in frail patients, because it often requires blood sampling from an arterial catheter. A widely used alternative to arterial sampling is SVCA, which identifies the voxels with minimal specific binding in the PET images, thus extracting a pseudo-reference region for non-invasive quantification. Unlike other reference region approaches, SVCA does not require specification of an anatomical reference region a priori, which alleviates the limitation of TSPO contamination in anatomically-defined reference regions in individuals with underlying inflammatory processes. Furthermore, SVCA can be applied to any TSPO PET tracer across different neurological and neuropsychiatric conditions, providing noninvasivequantification of TSPO expression.

**Methods:**

We provide an overview of the development of SVCA as well as step-by-step instructions for implementing SVCA with suggestions for specific settings. We review the literature on SVCAapplications using first- and second- generation TSPO PET tracers and discuss potential clinically relevant limitations and applications.

**Conclusions:**

The correct implementation of SVCA can provide robust and reproducible estimates of brain TSPO expression. This review encourages the standardisation of SVCA methodology in TSPO PET analysis, ultimately aiming to improve replicability and comparability across study sites.

## Introduction

The need for arterial blood sampling to quantify the binding potential of positron emission tomography (PET) tracers is a major obstacle to the widespread use of PET in research protocols, let alone in clinical practice. 18 kDa translocator protein (TSPO) PET imaging is no exception. In recent years, several approaches have been proposed as less invasive alternatives to arterial sampling; these include image-derived input function [1], population-derived input function [2], venous-based input function [3, 4] and simultaneous estimation (SIME) of the input function [5, 6]. These methods, however, have been unable to provide reliable and replicable solutions that are consistently valid across tracers and conditions.

For TSPO PET studies in particular, these approaches have been unable to account for the complex interactions between tracers and TSPO sites in the blood and vascular compartments [7, 8]. In addition, the ubiquitous expression of TSPO in the brain precludes modelling with a reference region approach. Thus, some authors have proposed using a pseudo-reference (or normative) region—that is, a region that expresses TSPO but whose density does not change under pathological conditions. Examples of pseudo-reference regions include the cerebellum [9], the occipital cortex [10, 11], the white matter [12] or even the whole brain [13]. Although normalizing over the whole brain may improve the detection of focal effects by robustly reducing between-subject variability, it also precludes the possibility of detecting global effects. This becomes problematic when the condition investigated is characterized by widespread, rather than regional, inflammation (e.g. neurologic disorders with widespread inflammation, peripheral inflammatory challenges).

To overcome the lack of a proper reference region, Turkheimer and colleagues described a supervised clustering algorithm (SVCA) to identify a reference region without prior anatomical hypothesis [14]. SVCA uses pre-defined kinetic classes to segment the tissue and to automatically extract reference time-activity curves, defined as the average curve of all voxels where the specific binding component is minimal. This algorithm, initially validated for ^11^C-(*R*)-PK11195, allows for the non-invasive quantification of TSPO across a variety of diseases. The method has been replicated by independent groups [15] and used in different clinical conditions associated with microglial activation, including Alzheimer’s disease [16, 17], traumatic brain injury [18], psychosis [19] and glioma [20]. However, ^11^C-(*R*)-PK11195 is a radioligand with low specific binding [21], and there seems to be an inverse relationship between the affinity of the tracer and the effectiveness of SVCA [22]. The method has been extended to other TSPO tracers including ^18^F-DPA-714 [23] and ^11^C-PBR28 [24], two second-generation tracers with ~ 1.5-fold and ~ 5–6-fold higher affinity to TSPO than ^11^C-(*R*)-PK11195, respectively [25].

The technical implementation of SVCA is not standardized, which may hamper the reproducibility of the results. Definition of kinetic classes, noise, quality of raw data, use of masking, normalization in the image processing and the effects of genetic polymorphism of TSPO ligands [26] all make it difficult to compare results obtained with the SVCA method. This paper seeks to function as a practical guide for SVCA implementation in the context of TSPO PET studies. The paper focuses on explaining the most common choices when implementing the method and highlights its main limitations.

## The supervised clustering approach

SVCA aims to identify voxels that exhibit a kinetic profile similar to those of the low-binding grey matter in healthy volunteers. These voxels can be used as a pseudo-reference region because they are less affected by inflammation and contain a minimal amount of TSPO. The main interest of SVCA would be to extract these voxels in participants who are affected by global neuroinflammatory processes.

SVCA first requires that a set of kinetic classes representing the expected tracer kinetics in different tissue types be defined, including both reference and non-reference tissue. Defining the kinetic classes is the most laborious and time-consuming step of the SVCA, but it needs to be performed only once for a given tracer and scanner combination.

Once the set of kinetic classes is built, the SVCA will calculate a set of weights for each PET scan voxel, with one weight corresponding to each kinetic class. The weight assigned to a tissue kinetic class represents how much that class contributes to the observed activity of the given voxel. Only voxels with high relative weight for the reference region tissue class are included to calculate the final reference curve.

A description of the two steps of the SVCA is detailed below. The first specifies how the kinetic classes are defined, and the second describes how reference region voxels are extracted. It should be noted that the SVCA procedure described here is specific to TSPO tracers, although the same rationale can be applied to all PET tracers.

### Part 1: Defining the kinetic classes

**Fig. 1:**
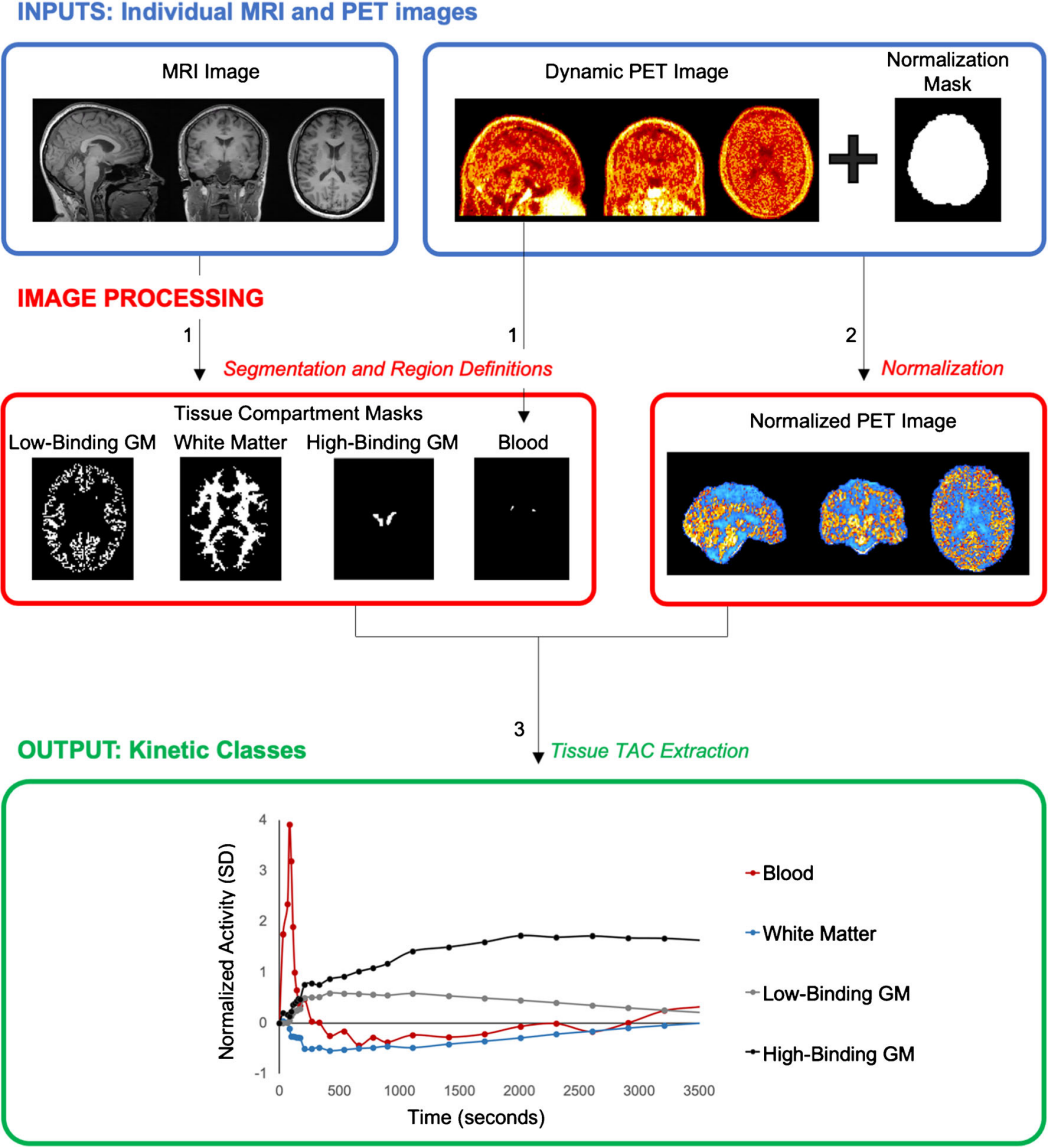
Image processing for the definition of kinetic classes. (1) Tissue compartment masks are defined using tissue segmentation and region definitions from structural MRI. Thalamus is shown as an example of high-binding grey matter (GM) tissue, while a combination of both PET and MRI images can be used to extract the blood kinetic class. (2) Voxels of dynamic PET contained within the normalization mask are used for PET normalization. (3) Normalized time-activity curves (TACs) are extracted from normalized dynamic PET for each tissue compartment mask

Defining the kinetic classes first requires a dataset of healthy volunteers (*training dataset*, generally *N* > 10) acquired with the same tracer and, ideally, with the same experimental protocol and scanner as the PET images to which the SVCA will be applied (*testing dataset*). Good quality anatomical MRI images are also necessary to define tissue masks. The data processing pipeline performed for each subject of the training dataset is summarized in Fig. 1.

The dynamic PET images of each participant must first be normalized in order to enhance the dissimilarity of the tracer kinetics between tissue types and to reduce intra-subject variability. PET image normalization is done by subtracting the mean of all voxels within the PET frame from each voxel of that frame and dividing the result by the standard deviation of all voxels within the PET frame. Because it has been shown to improve SVCA results [15, 27], the use of a brain mask in this step (i.e. using the mean and standard deviation of only the voxels within the brain mask rather than the whole frame) is recommended.

The kinetic classes are extracted from the normalized dynamic PET images. Generally, for TSPO PET applications, four different classes are defined: (1) low-binding grey matter; (2) white matter; (3) blood and (4) high-binding grey matter or a region with high specific binding. The low-binding grey matter class is assumed to represent the expected reference region kinetics. These classes are defined as follows:

1. Low-binding grey matter can be derived from the cerebellar and/or cerebral cortical grey matter regions in healthy volunteers. These regions can be obtained by segmenting the corresponding anatomical MRI into different tissue types and aligning the grey matter mask to the PET image. To limit partial volume effects, only voxels with a high probability (generally > 0.9) of being grey matter should be included and, in the case of a non-probabilistic segmentation, a small erosion should be applied to the final mask.
2. White matter can similarly be derived from the supratentorial white matter region in healthy volunteers. Cerebellar white matter is usually smaller and more difficult to segment on anatomical MRI. Partial volume effects can again be limited by only including voxels with a high probability of being white matter or by eroding the non-probabilistic white matter mask.
3. Blood activity can be derived by summing the first frames of the dynamic acquisitions (usually the first 2 to 3 min) and by extracting the voxels with the highest PET signal (usually ~ 50). Other methods consist of segmenting the carotids or the cerebral sinuses directly from the summed PET or MRI images. The final shape of the blood activity curve should be visualized to check that it exhibits the expected behaviour of a sharp peak at early frame times followed by a fast washout, typical of PET blood input functions.
4. High-binding grey matter can be derived from the pathological grey matter segmented in a set of participants with known neuroinflammatory processes. Alternatively, the high-binding grey matter can be derived from the thalamic region in healthy volunteers, as this is the region with the highest expression of TSPO in the healthy brain [23, 28].

The final kinetic classes are obtained by averaging all the normalized time-activity curves across participants. Alternatively, the median can be used, which may be more appropriate when handling data outliers. As an additional note, in neuroinflammation PET studies with second-generation TSPO tracers, the Ala147Thr genetic polymorphism modulates the tracer-to-target affinity. Therefore, separate sets of kinetic classes for high- and medium-affinity binders (HABs and MABs, respectively) should be created for each subgroup of participants with a similar tracer binding profile.

### Part 2: Extracting the reference region

To extract a reference region with the SVCA approach, the dynamic PET scan must first be normalized as described in part 1, above. It is crucial that the same definition of brain mask as the one used for generating the kinetic classes is employed for this step. Failing to use the same mask will lead to a mismatch between testing and training data which could lead to the incorrect selection of voxels to be used as the final reference region.

The second step consists of mathematically comparing each voxel of the normalized PET scan to the kinetic classes generated in part 1. More specifically, a multilinear regression is performed where the response variable is the time-activity curve of a single voxel from the normalized PET scan and the explanatory variables are the pre-defined kinetic classes (see Eq. (1), multilinear regression model). In other words, the normalized time-activity curve of each voxel is represented as a weighted linear combination of the kinetic classes.

1 $$ {\mathrm{TAC}}_v={w}_1{K}_1+\dots +{w}_i{K}_i+\dots +{w}_n{K}_n\ \left({w}_1,\dots, {w}_i,\dots, {w}_n\ge 0\right) $$

The equation is solved using a non-negative linear estimator that provides the weights (*w*_*i*_) for each kinetic class (*K*_*i*_) that give the best description of the voxel time-activity curve (TAC_*v*_). These weights can be thought of as the estimated contribution of each corresponding kinetic class to the activity of the voxel under investigation. To reduce the computational cost and prevent the selection of voxels of unwanted tissue types, the calculation of the weights can be limited to low-binding grey matter voxels rather than the full brain mask.

The ratio of the contribution of the low-binding grey matter class (*w*_LBGM_) to the sum of the contribution of all classes ($\sum \limits_1^n{w}_i$) is calculated (see Eq. (2), calculation of low-binding grey matter ratio (LBGM ratio)) and used to determine whether the voxel should be included as a reference voxel.

2 $$ \mathrm{LBGM}\ \mathrm{ratio}=\frac{w_{\mathrm{LBGM}}}{\sum \limits_1^n{w}_i} $$

**Fig. 2:**
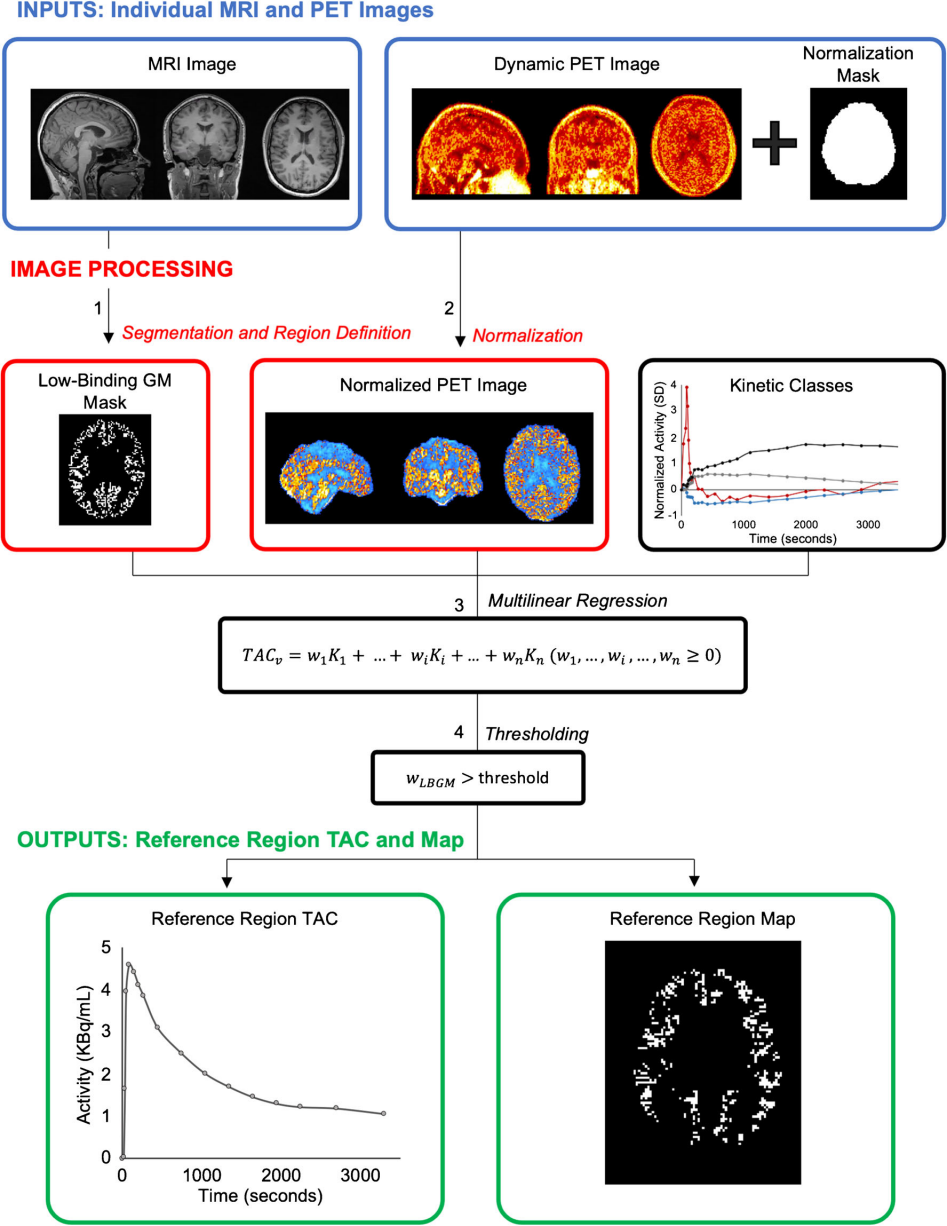
Extraction of the voxels for the supervised reference region. (1) Low-binding grey matter (GM) mask containing prospective reference region voxels is defined using tissue segmentation and region definition from structural MRI. (2) Voxels of dynamic PET contained within the normalization mask are used for PET normalization. (3) Multilinear regression is performed on each voxel of the normalized dynamic PET image within the prospective reference region mask, providing weights (*w*_*i*_) for each kinetic class (*K*_*i*_) that give the best description of the voxel time-activity curve (*TAC*_*v*_). (4) Voxels with a low-binding grey matter tissue class weight (*w*_LBGM_) greater than a pre-defined threshold are selected to be used as reference

Only voxels with a low-binding grey matter ratio greater than a pre-defined threshold (usually > 0.9) are included in the final mask in order to only include highly homogenous grey matter voxels while maintaining a sufficient number of voxels to use as reference [29]. The reference region time-activity curve is obtained by averaging the time-activity curves of the voxels included in the final mask. The pipeline for extracting the reference region is summarized in Fig. 2.

### Quality control

It is important to run quality control steps throughout the SVCA to ensure that the kinetic classes, selected reference region voxels and final SVCA reference region time-activity curve exhibit their expected behaviour. First, the normalized time-activity curves for the training dataset should exhibit similar behaviour (i.e. time-activity curve shape) within each tissue type. Plotting the normalized time-activity curves of the individual tissue classes against each other is important for identifying tracer kinetic heterogeneity and investigating outliers, both of which must be done before calculating the summary statistic to be used as the kinetic class.

Once the SVCA reference region has been extracted, the selection of voxels should be visualized to ensure that a suitable number of voxels are present and that these are generally evenly distributed across the healthy grey matter. If no or a negligible number of voxels have been selected, it means that SVCA likely failed to find a suitable reference region.

Finally, the time-activity curve of the extracted SVCA reference region can be plotted and compared with the time-activity curve of anatomically defined low-binding grey matter regions (e.g. cerebellar grey matter). The supervised reference region time-activity curve is expected to have a comparable or higher peak at early frame times and comparable or lower activity at later frame times than that of the anatomically defined low-binding grey matter reference region.

### Validation

SVCA must be validated before it can be applied for the first time to a dataset using a new PET tracer. Proper validation would ensure that the reference region extracted with SVCA lacks specific/displaceable binding. In this context, a blocking study could be conducted by applying the SVCA to the baseline scan to extract a candidate region and then by testing whether the distribution volume (*V*_T_) of this region is affected by the blocking agent [30]. However, given the background level of TSPO expression present in the brain of even healthy volunteers, some degree of specific binding will inevitably be present in the extracted region [31, 32]. Nonetheless, a blocking study would still be useful because it would allow the extent of TSPO expression in the extracted region to be quantified.

A less stringent requirement would be to verify whether the amount of specific binding included in the extracted reference region occurs independently of the condition under analysis. This could be done by simply comparing the *V*_T_ of the reference regions extracted in different clinical groups. It is important to note that failing to show a difference—for example by showing a non-significant *p* value—does not suffice; rather, the absence of a significant difference has to be demonstrated by appropriate statistical procedures (e.g. equivalence tests) [33].

### Code availability

An open-source Matlab-based implementation for SVCA is freely available at https://github.com/molecular-neuroimaging/svca.

## Applications in brain TSPO PET studies

Because of the obvious interest in avoiding arterial sampling, SVCA has been used in several clinical research protocols with, to date, three different TSPO tracers. An overview of these studies highlights not only the versatility of the technique but also the different methodological approaches and improvements in the implementation.

### ^11^C-(*R*)-PK11195

The SVCA method for 11C-(*R*)-PK11195 has been extensively used to study several neurological and psychiatric conditions, including Alzheimer’s disease [16, 17], multiple sclerosis [34], traumatic brain injury [18], schizophrenia [19] and changes in TSPO expression in normal ageing [35]. In Turkheimer’s initial implementation, the normalization step was performed at the whole field-of-view level, i.e. the entire PET frame was normalized by subtracting its mean and by dividing the result by its standard deviation. Moreover, six different kinetic classes were defined: nonspecific grey matter, nonspecific white matter, pathologic TSPO binding, blood pool, skull and muscle. All these classes were built from a cohort of 12 healthy volunteers who underwent an ^11^C-(*R*)-PK11195 PET scan, except for the pathologic TSPO binding class, which was defined on the striatum and globus pallidus of three patients with Huntington’s disease. The nonspecific grey matter class was considered as the reference region. With this method, a reference region was extracted in six healthy volunteers for whom the arterial input function was also available. The binding potentials (*BP*_ND_, [36]) of several regions of interest (ROIs) in the brain were estimated using rank-shaping exponential spectral analysis [37] with either the arterial input function or the extracted reference region. Good agreement was observed between arterial input and reference tissue input-derived *BP*_ND_ (Pearson’s correlation = 0.81, *p* < 10^-5). This implementation of the SVCA was further tested on four patients with Alzheimer’s disease who underwent two ^11^C-(*R*)-PK11195 PET scans each, with a delay of less than 6 weeks between the two exams. The *BP*_ND_ estimated using the simplified reference tissue model (SRTM) obtained a mean intraclass correlation coefficient (ICC) of 0.88 and a mean test–retest variability of 10.6% across ROIs, suggesting good reproducibility of the method.

An improvement over the initial implementation was subsequently proposed by Boellaard and colleagues [27] and further validated by Yaqub and colleagues [15]. This method applied a brain mask to the PET exam before applying the SVCA method. This had the double advantage of avoiding the effects of differences in field-of-view between different scanners in the normalization step as well as requiring only four classes instead of six, as skull and muscle were not necessary anymore. In the method by Boellaard and colleagues [27], this optimized implementation (called SVCA4) was tested against the original (SVCA6) and used the cerebellum as the reference region in nine healthy volunteers and nine patients with Alzheimer’s disease who underwent a 60-min ^11^C-(*R*)-PK11195 PET exam. The arterial input function was available for all participants. Although few quantitative results were reported, the reference region extracted using SVCA4 appeared to have lower *V*_T_ values (estimated via a two-tissue compartment model (2TCM)) than the reference region extracted using SVCA6 or the cerebellum, suggesting that it contained less specific binding. Notably, *V*_T_ of the reference region extracted with SVCA4 was statistically different between young healthy volunteers and patients with mild cognitive impairment and Alzheimer’s disease, indicating contamination by specific binding in patient groups. Moreover, thalamic *BP*_ND_ estimated using SRTM with the reference region extracted with SVCA4 appeared to correlate better with those obtained using 2TCM with arterial input function and, on average, was higher than *BP*_ND_ obtained with SRTM with the reference region extracted with SVCA6 or the cerebellum. These results were essentially confirmed in a cohort of nine young healthy volunteers, eight old healthy volunteers, nine patients with mild cognitive impairment and eight patients with Alzheimer’s disease who underwent a 60-min ^11^C-(*R*)-PK11195 PET exam with measurement of arterial input function [15]. Both *V*_T_ and blood fraction (*V*_b_) estimated with 2TCM were generally lower in the reference region extracted using SVCA4 compared to that extracted using SVCA6 or the cerebellum, indicating lower levels of blood volume and specific binding. Moreover, significant differences between clinical groups in thalamic *BP*_ND_ estimated with SRTM were observed only when using the reference region extracted with SVCA4 or SVCA6 but not with the cerebellum.

Another study by Plavén-Sigray and colleagues assessed SVCA4 performance in a group of six healthy volunteers who underwent two 60-min ^11^C-(*R*)-PK11195 PET scans each with a delay of approximately 6 weeks between the two scans [38]. An arterial input function was collected for all participants. The authors found poor reproducibility (ICC < 0.5) of *BP*_ND_ estimates regardless of the method used to calculate it (i.e. 2TCM with arterial input function, SRTM with reference region extracted with SVCA4 or with cerebellum). Moreover, large differences in magnitude and poor-to-non-existent correlations between the *BP*_ND_ values derived with arterial input function and those derived with reference regions were observed. The impact of different kinetic classes, derived from three different groups of participants acquired in different locations and with different scanners, was also evaluated. *BP*_ND_ estimates were highly correlated between different kinetic classes, albeit with significant differences in terms of absolute values. The authors imputed the low repeatability of *BP*_ND_ to the fact that the cohort comprised young healthy volunteers only (mean age = 25.8 ± 3.9 years); TSPO expression would be expected to be minimal across the brain in this cohort, and most of the ^11^C-(*R*)-PK11195 signal consists of nonspecific binding and unbound radioligand. In this context of high background signal and the small effect of interest, the estimated *BP*_ND_ values were close to zero (or even negative) and thus particularly sensitive to even small amounts of measurement error.

### ^18^F-DPA714

SVCA for ^18^F-DPA714 has already been used for clinical applications, notably in multiple sclerosis [39] and Parkinson’s disease (Lavisse et al., 2020 *in press*). Love and colleagues first proposed the implementation of SVCA for ^18^F-DPA714 [40]. In their study, eight healthy volunteers and two patients with stroke underwent a 60-min ^18^F-DPA714 PET exam; no blood samples were available. Four kinetic classes were defined as in the SVCA4 implementation for ^11^C-(*R*)-PK11195: blood, white matter, low specific binding grey matter from the healthy volunteers and specific binding grey matter from manually defined hyperintense voxels of the PET data of stroke patients. The authors compared the areas under the time-activity curves of the extracted reference regions against those of the cerebellum, which was assumed to be devoid of TSPO in both groups of participants and found no significant differences between reference regions. The authors also reported significant correlations between the *BP*_ND_ of the thalamus (estimated with SRTM2) calculated using either the SVCA or cerebellum references, albeit without reporting the magnitude of the correlation.

In a more extensive evaluation of SVCA for ^18^F-DPA714, 14 healthy volunteers (seven HABs and seven MABs) underwent a 90-min ^18^F-DPA714 PET exam [23]. Three participants (two HABs and one MAB) had a second PET scan around 1 week later, and 10 participants (seven HABs and three MABs) had data collected with an arterial input function. Four kinetic classes were defined: blood pool, white matter, low specific binding (corresponding to the cerebellum) and high specific binding (corresponding to the thalami). The four classes were derived from the ensemble of the population (SVCA_ALL) and for HABs and MABs separately (SVCA_HAB/MAB). *BP*_ND_ was estimated in several brain ROIs using 2TCM with arterial input function as well as Logan plot with reference region, where the reference region was extracted using SVCA_ALL, SVCA_HAB/MAB and the cerebellum. All reference regions yielded *BP*_ND_ estimates that correlated highly with those obtained with arterial input function (*r* > 0.9) and similarly good test–retest variability (< 7%). The reference regions extracted with both SVCA methods had lower *V*_T_ compared to the cerebellum which, in turn, resulted in higher and less variable estimates of *BP*_ND_ across different brain regions. Thalamic and cingulate cortex differences in *BP*_ND_ between HABs and MABs were observed only when the reference region extracted with SVCA_ALL and SVCA_HAB/MAB was used, and not when the cerebellum was used. In general, SVCA_ALL and SVCA_HAB/MAB gave similar results, even though *BP*_ND_ estimates obtained with SVCA_ALL were closer to those obtained with arterial input function and had a lower relative error rate than those obtained with SVCA_HAB/MAB [23].

SVCA for ^18^F-DPA714 has also been validated in a non-human primate model of local neuroinflammation in which three-dimensional TSPO immunohistochemistry was performed post-mortem after the PET exam [41]. Four kinetic classes were derived as in the study by García-Lorenzo and colleagues [23] from five unrelated non-human primates who underwent a 90-min ^18^F-DPA714 PET scan. A voxel-wise *BP*_ND_ map of the non-human primate under analysis was estimated with Logan plot using both the reference region extracted with SVCA and the cerebellum. These values were used to build a binary classifier of TSPO positive and negative voxels, where the true reference value was determined based on post-mortem TSPO immunohistochemistry. The *BP*_ND_ estimated using the SVCA approach performed slightly better than that estimated using the cerebellum as a reference region (area under the curve of the receiver operating characteristic curve = 0.92 vs 0.90).

Finally, a variant of SVCA for ^18^F-DPA714 was developed for imaging in rodents [42]. The algorithm consisted of only three classes (brain tissue with low specific binding; brain tissue with high specific binding and extracerebral tracer signal), and it was modified to be used at a region level rather than at a voxel level. This method was tested on 25 Wistar rats for which neuroinflammation was induced by injecting lipopolysaccharide into the right striatum. *BP*_ND_ was estimated using SRTM with either the reference region extracted by the modified SVCA or the contralateral striatum, which was considered the gold standard. The *BP*_ND_ values estimated with the two methods were highly correlated (*r* > 0.9), suggesting that this variant of SVCA can also be used in animal models of diffuse inflammation where an a priori reference region is not known.

### ^11^C-PBR28

Zanotti-Fregonara and colleagues first explored SVCA for ^11^C-PBR28 in a study of 21 healthy volunteers, 11 patients with mild cognitive impairment and 25 patients with Alzheimer’s disease who underwent a 90-min ^11^C-PBR28 PET exam with measurement of both arterial input function and free fraction in plasma (*f*_p_) [24]. Four kinetic classes were used: grey matter, normal white matter, sinus extracted from healthy volunteers and pathologic grey matter extracted from the voxels of the inferior parietal and middle and inferior temporal cortices in which ^11^C-PBR28 binding was significantly increased in patients with Alzheimer’s disease. Distribution volume ratio (DVR) estimated with Logan reference region and *V*_T_/*f*_p_ estimated with 2TCM with arterial input function were used to test differences between the three groups of participants. DVR estimates were substantially less variable than *V*_T_/*f*_p_ estimates (coefficient of variation = 2%–11% for DVR and 13%–36% for *V*_T_/*f*_p_), resulting in greater sensitivity to detect regional abnormalities in the brains of patients with Alzheimer’s disease.

## Discussion

As reviewed above, SVCA is a widely used alternative to arterial sampling that identifies the voxels with minimal specific binding in PET images, thus extracting a pseudo-reference region for non-invasive quantification. This approach is particularly important because, despite the absence of brain tissues or regions with no or negligible TSPO expression, reference region approaches have, to date, been the most widely used quantification methods for TSPO PET neuroinflammation studies. These pseudo-reference approaches are simple and can only return indirect metrics of TSPO distribution in the brain. Quantification with full kinetic modelling and arterial input functions are extremely challenging for clinical applications because of their invasive nature and the need for sophisticated equipment and experienced personnel (e.g. anaesthesiologists for placing the arterial catheter and lab specialists to analyse the blood samples [43]). Furthermore, absolute quantification of TSPO tracer binding with or without normalization by a plasma-free fraction is associated with high variability that may be attributable to challenges in obtaining accurate blood measurements and modelling the brain kinetics of TSPO tracers [44] in addition to physiologic variability [45]. Notably, the sparse amount of data in the literature of SVCA and the heterogeneity of its applications to clinical data sometimes hinder a comprehensive assessment of the strengths and weaknesses of SVCA in different clinical situations.

### Implementation aspects

As summarized in Table 1, different SVCA studies have implemented this method in different ways. In particular, different masking methods have been used for the normalization step, including whole head mask (brain, skull and muscle), whole brain mask (brain only) or simply using the entire frame without masking. Entirely skipping a masking step in the normalization incorporates additional background noise into the mean and standard deviation calculations, which may contribute to the shape of the kinetic classes in an unpredictable way. Moreover, if tissues such as muscle and bone outside the brain are included in the mask, additional classes must be included in the kinetic class definitions, which increases variability because additional parameters have to be estimated. Our recommendation is to limit the number of kinetic classes by using a brain mask (i.e. by excluding the skull and other non-brain tissue) and to use the same brain mask (i.e. calculated using the same method) for dynamic PET normalization during the definition of the kinetic classes and reference region extraction.

**Table 1:** SVCA implementation in TSPO PET studies

Tracer	Normalization mask	N of classes	High-binding class	Reference class	Main results	Ref
^11^C-(*R*)-PK11195	Whole frame	6	Striatum and globus pallidus of patients with Huntington’s disease	Grey matter of healthy volunteers	+ high correlations with the arterial input function + good test–retest reproducibility in patients with Alzheimer’s disease	[14]
^11^C-(*R*)-PK11195	Grey and white matter	4	Grey matter of patients with traumatic brain injury	Grey matter of healthy volunteers	+ low specific binding in the extracted reference region + high sensitivity to clinical abnormalities + highly correlated results when using different sets of kinetic classes − differences in the *V*_T_ of the extracted reference region between clinical subgroups − poor test–retest reproducibility and low correlation with arterial input function in healthy volunteers	[15, 27, 38]
^18^F-DPA714	Whole brain	4	Hyperintense voxels in patients with stroke	Grey matter of healthy volunteers	+ comparable results with the cerebellum	[40]
^18^F-DPA714	Whole brain	4	Thalamus of healthy controls	Cerebellar grey matter of healthy volunteers	+ low specific binding in the extracted reference region + high correlations with the arterial input function + good test–retest reproducibility in healthy volunteers (*N* = 3) + low variability of tissue estimates + high sensitivity to genotype differences	[23]
^11^C-PBR28	Whole brain	4	Inferior parietal and middle and inferior temporal cortices in patients with Alzheimer’s disease	Grey matter of healthy volunteers	+ low variability of the distribution volume ratio (DVR) estimates + high sensitivity to clinical abnormalities − differences in the time-activity curves of the extracted reference region between genotype subgroups	[24]

Defining kinetic classes has also varied between studies. For example, the low-binding grey matter class has been defined from the cortical grey matter, cerebellar grey matter or whole brain grey matter; the blood class has been manually or automatically derived and the high-binding class has been defined in a variety of ways in each study (Table 1). Some studies have also accounted for partial volume effect by eroding the tissue masks or by choosing only voxels with a high probability of belonging to a tissue type. Our recommendation is to account for partial volume effects in white matter and grey matter classes and to derive the high-binding class from patients known to be affected by neuroinflammatory processes, if available. Although no direct investigations have yet to be performed to assess the effect of using inflamed tissue from a patient cohort to define the high-binding kinetic class, SVCA has been successfully implemented in cancer where the tissue kinetics of patients is dramatically different from that of healthy controls [46]. The successful implementation of SVCA in cancer suggests that applying the normalization step to cohorts with different neuroinflammatory topology is not a major issue.

Other differences in implementation include how the low-binding grey matter ratio threshold is chosen and how the reference region time-activity curve is calculated (by simple or weighted average, where the weights used are those derived for the low-binding grey matter class by SVCA), as well as other details; presently, no specific recommendations for these aspects can be given.

### Portability of kinetic classes

The portability of the kinetic classes to data acquired from different imaging sites and with different experimental designs has not yet been systematically investigated. In [38], the 11C-(R)-PK11195 BP_ND_ estimates obtained with different sets of kinetic classes, derived from different sites, were highly correlated but different in terms of absolute value. This indicates that the use of kinetic classes derived from different imaging protocols will probably introduce a bias in the estimates, which may not be a problem if the bias is constant for all participants. In a non-TSPO brain PET study with ^11^C-PIB [47], the kinetic classes were defined from a dataset of healthy individuals and patients with Alzheimer’s disease acquired with ECAT EXACT HR+ (CTI/Siemens) [29] and then applied to an independent dataset of healthy volunteers and patients with multiple sclerosis acquired with a Siemens HRRT. The results supported the kinetic class portability, although performance was dependent on the PET reconstruction settings.

Our recommendation in this case is to re-define the kinetic classes using site-specific control data collected independently from but with the same PET imaging protocol as the study of interest. If the dataset of healthy volunteers is part of the study, the reference region in these participants should be obtained using a leave-one-out approach: for each participant in the healthy volunteer group, SVCA should be applied using the kinetic classes defined from all the other healthy volunteers except the one to which the method is applied.

### Anatomical reference region vs SVCA

Both anatomical and SVCA approaches have been extensively used to identify the reference tissues to be used for kinetic modelling, although these approaches vary across TSPO tracers and clinical applications. Anatomical-based reference region methods use prior knowledge of brain biology to identify those tissues where TSPO density does not change across conditions. Using this method, for example, brain regions unaffected by the disease can be identified in patient-control cross-sectional studies. Though such an approach might hypothetically be possible for focal inflammatory diseases, it would certainly not be possible in cases where neuroinflammation is expected to span across the entire brain parenchyma. In these cases, data-driven reference methods like SVCA are better alternatives for controlling the reference region-specific signal because they use statistical (and not biological) criteria to identify voxels with minimal TSPO density. Indeed, reference regions extracted with SVCA appear to contain less binding [15, 23, 27] and less blood volume fraction [15, 27] compared to anatomically defined reference regions, suggesting that SVCA derives more accurate reference regions.

Nonetheless, other elements must be considered before using SVCA. One key example is radioligand affinity to TSPO. In particular, tracer affinity modulates the contrast between tissue kinetics by increasing both parenchymal and vascular binding which, in turn, affects the use of cluster analysis to determine a pseudo-reference region [22]. When the contrast between grey matter and white matter kinetics was compared across different TSPO tracers, ^11^C-PBR28 was found to display the lowest tissue contrast, whereas middle-affinity tracers such as ^18^F-DPA714 appear to be amenable to the supervised definition of a reference region [22]. This result suggests that SVCA reference tissue analysis is poorly suited to high-affinity TSPO tracers, although further studies are needed to confirm this hypothesis.

### Presence of specific binding in the reference region

Given the background level of TSPO present everywhere in the brain, even in healthy volunteers, the presence of specific binding in the reference region is unavoidable. This specific binding is known to introduce a bias [48], but this is generally accepted for TSPO tracers [9, 49]. Advanced methods exist for accounting for this bias [13, 50], but these are rarely used. Moreover, it seems that some differences in the amount of specific binding present in the extracted reference regions are present across different clinical conditions [15, 24], which ultimately affects the sensitivity of the method to detect TSPO brain changes. As a result, the TSPO PET effect sizes obtained with SVCA are likely to underestimate the true magnitude of TSPO density changes in the study population. Nonetheless, the lower variability of estimates obtained with SVCA compared to those obtained with full arterial input function has the potential to increase statistical power, allowing the detection of more brain regions with increased TSPO expression.

## Conclusion

SVCA can be used to quantify TSPO PET imaging using a pseudo-reference approach that takes advantage of statistical, rather than anatomical, information about tracer kinetics to minimize the effects of reference region specific binding. Although SVCA has limitations, when implemented correctly, it provides robust and reproducible estimates of brain TSPO expression. Standardization of SVCA methodology in TSPO PET analysis is encouraged because it would improve the replicability of results and ease comparability across study sites.

## Declarations

### Disclaimer

The views expressed are those of the author(s) and not necessarily those of the NHS, the NIHR or the Department of Health.
